# Optimized permutation testing for information theoretic measures of multi-gene interactions

**DOI:** 10.1186/s12859-021-04107-6

**Published:** 2021-04-07

**Authors:** James M. Kunert-Graf, Nikita A. Sakhanenko, David J. Galas

**Affiliations:** Pacific Northwest Research Institute, 720 Broadway, Seattle, WA 98122 USA

**Keywords:** Permutation testing, Information theory, Multi-locus GWAS, Multivariable interactions

## Abstract

**Background:**

Permutation testing is often considered the “gold standard” for multi-test significance analysis, as it is an exact test requiring few assumptions about the distribution being computed. However, it can be computationally very expensive, particularly in its naive form in which the full analysis pipeline is re-run after permuting the phenotype labels. This can become intractable in multi-locus genome-wide association studies (GWAS), in which the number of potential interactions to be tested is combinatorially large.

**Results:**

In this paper, we develop an approach for permutation testing in multi-locus GWAS, specifically focusing on SNP–SNP-phenotype interactions using multivariable measures that can be computed from frequency count tables, such as those based in Information Theory. We find that the computational bottleneck in this process is the construction of the count tables themselves, and that this step can be eliminated at each iteration of the permutation testing by transforming the count tables directly. This leads to a speed-up by a factor of over 10^3^ for a typical permutation test compared to the naive approach. Additionally, this approach is insensitive to the number of samples making it suitable for datasets with large number of samples.

**Conclusions:**

The proliferation of large-scale datasets with genotype data for hundreds of thousands of individuals enables new and more powerful approaches for the detection of multi-locus genotype-phenotype interactions. Our approach significantly improves the computational tractability of permutation testing for these studies. Moreover, our approach is insensitive to the large number of samples in these modern datasets. The code for performing these computations and replicating the figures in this paper is freely available at https://github.com/kunert/permute-counts.

## Background

Genome-wide association studies (GWAS) have shed light on the genetics of complex traits and diseases, but single-locus analyses fail to detect the epistatic gene–gene interactions, which play a crucial role in the genetics of complex traits [1–3]. This has resulted in a proliferation of methods for detecting gene–gene interactions [4], for example: regression methods, including regularized regression techniques such as LASSO [5, 6]; ensemble methods such as random forests [7–9]; and multifactor dimensionality reduction [10, 11].

We focus here on the class of techniques based on information theory, which formulate entropy-based measures sensitive to multi-gene epistatic interactions. These approaches are powerful due to being inherently model-free and particularly sensitive to nonlinear relationships [3]. This has led to its own proliferation in entropy-based measures of epistatic interaction, including: Conditional Mutual Information [12], Information gain [13–18], Relative Information Gain [16, 19–21], Total Correlation [22–27], Synergy [28, 29], and the Information Delta [30, 31].

Though these different formulations vary, they share many of the same advantages inherent to the information theory-based approach, but also many of the same weaknesses, and of particular note here is the recurrent difficulty in constructing statistical tests for the significance of a detected interaction. There is typically no simple analytic formulation for the null distributions of these estimators, and thus significance tests require either some approximation or, more reliably, permutation testing. Permutation testing is often considered the “gold standard” for multi-test significance analysis [32, 33], and is the approach utilized by the majority of the above studies [20–27, 29, 34, 35].

Even in a single-locus GWAS, permutation testing is computationally costly [33]. SNP arrays may contain hundreds of thousands of individual SNPs, and thus there are hundreds of thousands of pairwise SNP-phenotype relationships to be tested. Higher-order relationships quickly lead to computationally intractable problems: this same number of SNPs leads to billions of possible three-way SNP–SNP-phenotype interactions, and to tens of trillions of four-way SNP–SNP-SNP-phenotype interactions. Detecting and testing these interactions becomes difficult on both statistical and computational levels.

In its simplest naive form, permutation testing consists of iterations of randomly permuting the phenotype labels and re-running the analysis pipeline. However, this approach can be optimized considerably, especially when performed multiple times: for example, standard packages such as PLINK [5] by default use an adaptive approach, which iteratively checks if the permutations already performed are sufficient to rule any of the observed SNP-phenotype associations as statistically insignificant, and drops insignificant SNPs from subsequent computations. Even the cost of this approach can be further reduced by an order of magnitude, and there exist multiple approaches for optimizing these single-locus analyses, including PRESTO [36], SLIP and SLIDE [37], and PERMORY [38].

In this paper, we develop an approach which reduces the computational cost of permutation tests by orders of magnitude for all information theory based measures. We identify the construction of count tables as the largest computational bottleneck, and devise a method for directly transforming these count tables to replicate a permutation test, without having to reconstruct them. We find that this reduces the computation time of each permutation by over three orders of magnitude. This approach therefore allows for the principled assessment of statistical significance in a multi-SNP association study, and enables the consideration and comparison of multiple candidate measures for multivariable dependence.

## Results

### Construction of count tables

Genotype and phenotype data can be represented with an $n\times m$ genotype array *G* and a length-*n* phenotype vector *p*, where *m* SNPs are measured for *n* individuals. The number of three-way SNP–SNP-phenotype interactions is typically quite large, as this scales as $m^2$. In this case of large *n* and *m*, we find that the bulk of the computation consists of merely computing the count tables for each possible tuple.

The computation of the joint entropy between the variables in a given tuple first requires the calculation of a count table. Consider a tuple consisting of two SNPs and a single phenotype. Each SNP takes a value 0, 1, 2 (for homozygous major, heterozygous, and homozygous minor alleles respectively), and the phenotype is binary with possible values 0 and 1. The count table *C* is then a $3\times 3 \times 2$ array:

1 $$\begin{aligned} C = \left[ \begin{bmatrix} c_{000} &{} c_{010} &{} c_{020}\\ c_{100} &{} c_{110} &{} c_{120}\\ c_{200} &{} c_{210} &{} c_{220}\\ \end{bmatrix} , \begin{bmatrix} c_{001} &{} c_{011} &{} c_{021}\\ c_{101} &{} c_{111} &{} c_{121}\\ c_{201} &{} c_{211} &{} c_{221}\\ \end{bmatrix} \right] , \end{aligned}$$

where $c_{ijk}$ is the number of individuals for whom the first SNP has a value of *i*, the second SNP has a value of *j*, and the phenotype has a value of *k*. Clearly, the elements sum to the total number of individuals *n*; dividing this array by *n* gives the joint probability estimates, from which the various joint entropies can be calculated, which can then be used to calculate information-theoretic measures for the corresponding tuple.

### Notation and reasoning

A count table *C* must be constructed for each of the billions of tuples. A naive approach to permutation testing would simply randomly shuffle the phenotype vector *p* and repeat the entire analysis, including the reconstruction of count tables from the data. We instead seek a transformation which, starting from a count table *C*, will generate a randomized count table $C^*$ from the same distribution of randomized count tables given by naive permutation. The first crucial observation is that the sum over the third axis of *C* will remain constant over a permutation test:

2 $$\begin{aligned} c_{ij0}+c_{ij1}=c^*_{ij0}+c^*_{ij1}\equiv n_{ij} \end{aligned}$$

where $n_{ij}$ is the number of individuals for whom the first SNP is *i* and the second SNP is *j*. With this notation, we can write:

3 $$\begin{aligned} C^* = \left[ \begin{bmatrix} c^*_{000} &{} c^*_{010} &{} c^*_{020}\\ c^*_{100} &{} c^*_{110} &{} c^*_{120}\\ c^*_{200} &{} c^*_{210} &{} c^*_{220}\\ \end{bmatrix} , \begin{bmatrix} n_{00}-c^*_{000} &{} n_{01}-c^*_{010} &{} n_{02}-c^*_{020}\\ n_{10}-c^*_{100} &{} n_{11}-c^*_{110} &{} n_{12}-c^*_{120}\\ n_{20}-c^*_{200} &{} n_{21}-c^*_{210} &{} n_{22}-c^*_{220}\\ \end{bmatrix} \right] , \end{aligned}$$

We need only compute the $k=0$ layer of this array, from which the $k=1$ layer immediately follows. We also have the constraint that:

4 $$\begin{aligned} \sum _{i,j}c_{ij0} = \sum _{i,j}c^*_{ij0} \equiv n_0 \end{aligned}$$

$n_0$ is the total number of individuals with phenotype label 0, which will also remain constant as the labels are shuffled.

With our notation and the constraints of Eqs. 2 and 4, we can begin to consider the effect of a permutation test upon a count table. Firstly, how is $c_{000}^*$ distributed? Consider the $n_{00}$ individuals with this genotype. If we randomly shuffle the phenotype labels, we are, in effect, randomly drawing without replacement $n_{00}$ labels from the population of *n* labels, $n_0$ of which have a value of 0. This process of drawing from a finite set of labels without replacement is described by the hypergeometric distribution, and we can write:

5 $$\begin{aligned} c^*_{000} \sim \text {Hypergeometric}(n,n_0,n_{00}) \end{aligned}$$

from which $c^*_{001}=n_{00}-c^*_{000}$ immediately follows.

When computing the next element, we must consider that the previous step has already assigned $n_{00}$ labels, $c_{000}^*$ of which had a value of 0. We again draw without replacement $n_{10}$ labels, now from a total population of $n-n_{00}$ phenotype labels, of which $n_0-c_{000}^*$ have value 0:

6 $$\begin{aligned} c^*_{100} \sim \text {Hypergeometric}(n-n_{00},n_0-c^*_{000},n_{10}) \end{aligned}$$

The next element is drawn iteratively in the same manner:

7 $$\begin{aligned} c^*_{200} \sim \text {Hypergeometric}(n-(n_{00}+n_{10}),n_0-(c^*_{000}+c^*_{100}),n_{20}) \end{aligned}$$

This process is repeated until all of the elements have been assigned.

### Algorithm for transformed count tables

More formally, this count transformation process can be written as follows:

1. From the original count table $c_{ijk}$, compute the genotype counts $n_{ij}$, the value-0 phenotype count $n_0$, and the total phenotype count *n*.
2. Assign an (arbitrary) order to the indices (*i*, *j*). This will be the order in which the elements are assigned. For example, let:
  $$\begin{aligned} \{(i,j)\} = \{(0,0)< (1,0)< (2,0)< (0,1)< \cdots < (2,2)\} \end{aligned}$$
3. For each (*i*, *j*) in the ordered set, sample from the hypergeometric distribution:
  $$\begin{aligned} c^*_{ij0} \sim \text {Hypergeometric}\left( n-\sum \nolimits _{(i',j')<(i,j)}n_{i'j'},\quad n_0-\sum \nolimits _{(i',j')<(i,j)}c^*_{i'j'0},\quad n_{ij}\right) \end{aligned}$$
4. Calculate the corresponding number of counts with phenotype value 1:
  $$\begin{aligned} c^*_{ij1} = n_{ij} - c^*_{ij0} \end{aligned}$$

## Discussion

### Comparing generated distributions

To check that this method works as intended, we verified that the distribution of count tables generated via our method is indistinguishable from count tables generated by direct permutation of the phenotype labels. Specifically, we randomly generated a total of $N_P=1{,}000{,}000$ permuted count tables using each method, and found the distributions of the permuted elements $c^*_{ij0}$ to be both visually and statistically indistinguishable (via an ensemble of Epps–Singletons tests between the two distributions [39]). Further details on how these count tables were generated and how the analysis was performed are given in the Methods.

### Comparing computational complexity

**Fig. 1:**
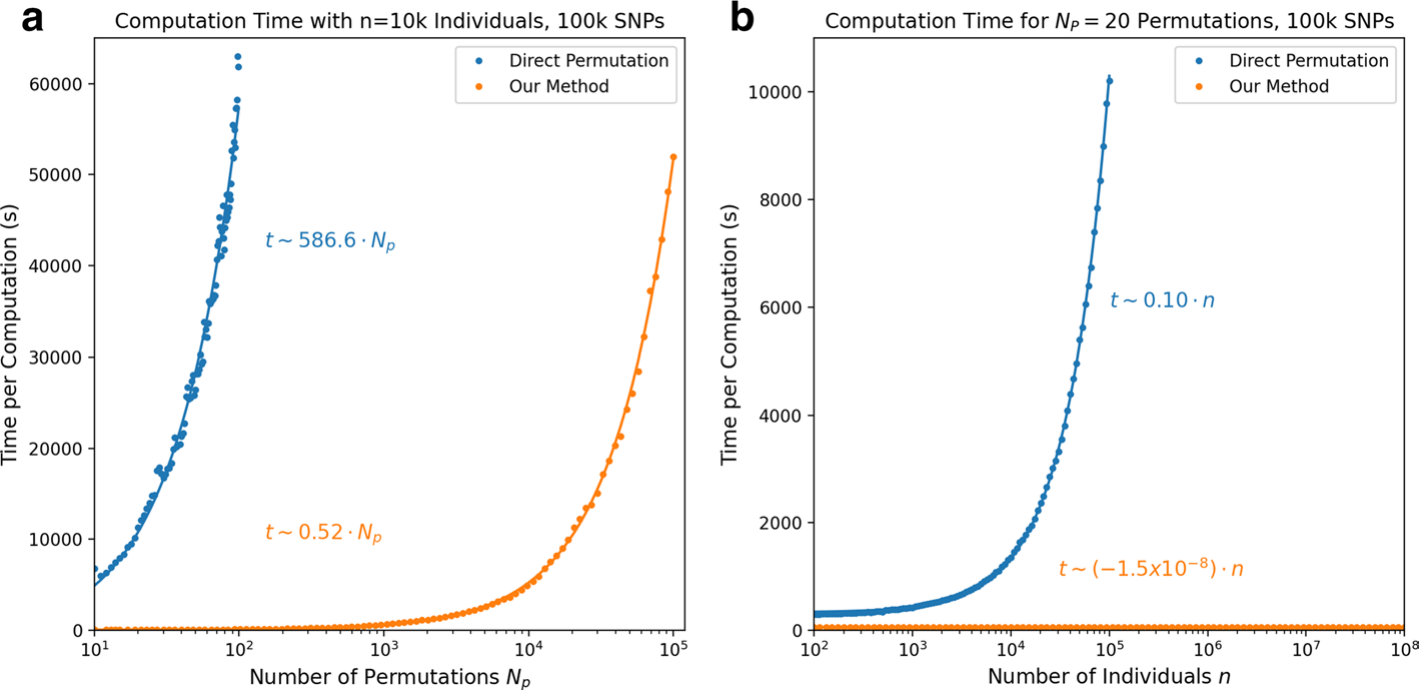
**a** Computation time as a function of the number of permutations $N_p$, for a synthetic dataset with a fixed number of individuals $n=10{,}000$ and 100 k SNPs. Both direct permutations (in blue) and our method (in orange) are $\mathcal {O}(N_p)$ (note that the horizontal axis is logarithmic, and the best fit lines plotted here are indeed linear). Our method is faster by a factor of over $10^3$ per permutation. **b** Computation time as a function of the number of individuals *n*, for a synthetic dataset with a fixed number of permutations $N_p=20$ and 100 k SNPs. Direct permutation is $\mathcal {O}(n)$ but our approach is $\mathcal {O}(1)$ (i.e. computation time does not depend on the number of samples for this approach)

We can also generate synthetic data (as described in the Methods) to compare the computational cost of each approach. Figure 1 compares the computational complexity of the naive direct permutation approach as compared to our method, as a function of both the number of individuals *n* and number of permutations $N_p$. In Fig. 1a, we calculate the computation time as a function of $N_p$, with a fixed $n=10{,}000$ samples and 100k SNPs. The computation time of both methods scales linearly with the number of permutations (i.e. they are both $\mathcal {O}(N_p)$). However, the linear fits to each method imply a time per permutation of 586.6*s* for the direct permutation method and 0.52*s* for our method. Our method is therefore over $10^3$ times faster for each permutation, for this number of samples.

Figure 1b, which calculates the computation time as a function of number of samples *n* with a fixed $N_p=20$, shows an even clearer computational advantage of our approach. The direct permutation approach scales linearly with the number of samples (i.e. it scales as $\mathcal {O}(n)$), whereas the computation time for our method does not depend on the number of samples (i.e. it scales as $\mathcal {O}(1)$). This is not unexpected, since our method bypasses the need to perform any operations on the original $n\times m$ array. This represents a considerable computational savings for datasets with a large number of samples.

## Conclusion

This paper outlines the algorithm for direct transformation of count tables, shows that the results are identical to those obtained by the naive approach of directly permuting the phenotype labels, and shows the considerable reduction in computational expense using this method. Specifically, we demonstrate a reduction of computation time per permutation by a factor of over $10^3$, and show that our method is insensitive to the total number of samples while the naive approach scales linearly. By bypassing the most computationally expensive step of the naive approach to permutation testing, our method therefore considerably decreases the cost of permutation testing for information theoretic measures.

Future developments on this method should incorporate additional methods for decreasing the computational cost of permutation analyses. For example, it is common for pairwise GWAS analyses to use an adaptive scheme which iteratively drops interactions if they are clearly not statistically significant (e.g. this is done by default in PLINK [5]). A similar adaptive scheme could be implemented here on top of our method.

Given the recent proliferation of large datasets for which multilocus analyses can yield novel biological insights, and given the importance of permutation testing for information theoretic measures without a clean analytically known null distribution, we believe that our approach is a valuable contribution towards making these large and important analyses more computationally tractable. The code for performing these computations and replicating the figures in this paper is freely available at https://github.com/kunert/permute-counts.

## Methods

### Synthetic dataset and its count distributions

Each SNP–SNP-phenotype tuple in our synthetic dataset is generated as described in this section. SNP data is generated independently for both SNPs by assuming perfect Hardy-Weinberg equilibrium with a minor allele frequency of $p = 0.45$ (i.e. we generate a $n\times 2$ genotype array where each element has a probability $p^2$ of being 0, probability $2p(1 - p)$ of being 1, and probability $(1-p)^2$ of being 2). We similarly generate a binary phenotype vector which has a probability $q=0.66$ of equaling zero. As we will establish later, the values of *p* and *q* do not affect our results.

The above parameters lead to a random count table such as the one below, generated for $n=10{,}000$ individuals:

8 $$\begin{aligned} C = \left[ \begin{bmatrix} 619&{}992&{}439\\ 964&{}1576&{}614\\ 409&{}674&{}264\\ \end{bmatrix} , \begin{bmatrix} 347&{}527&{}200\\ 496&{}862&{}332\\ 220&{}328&{}137\\ \end{bmatrix} \right] , \end{aligned}$$

**Fig. 2:**
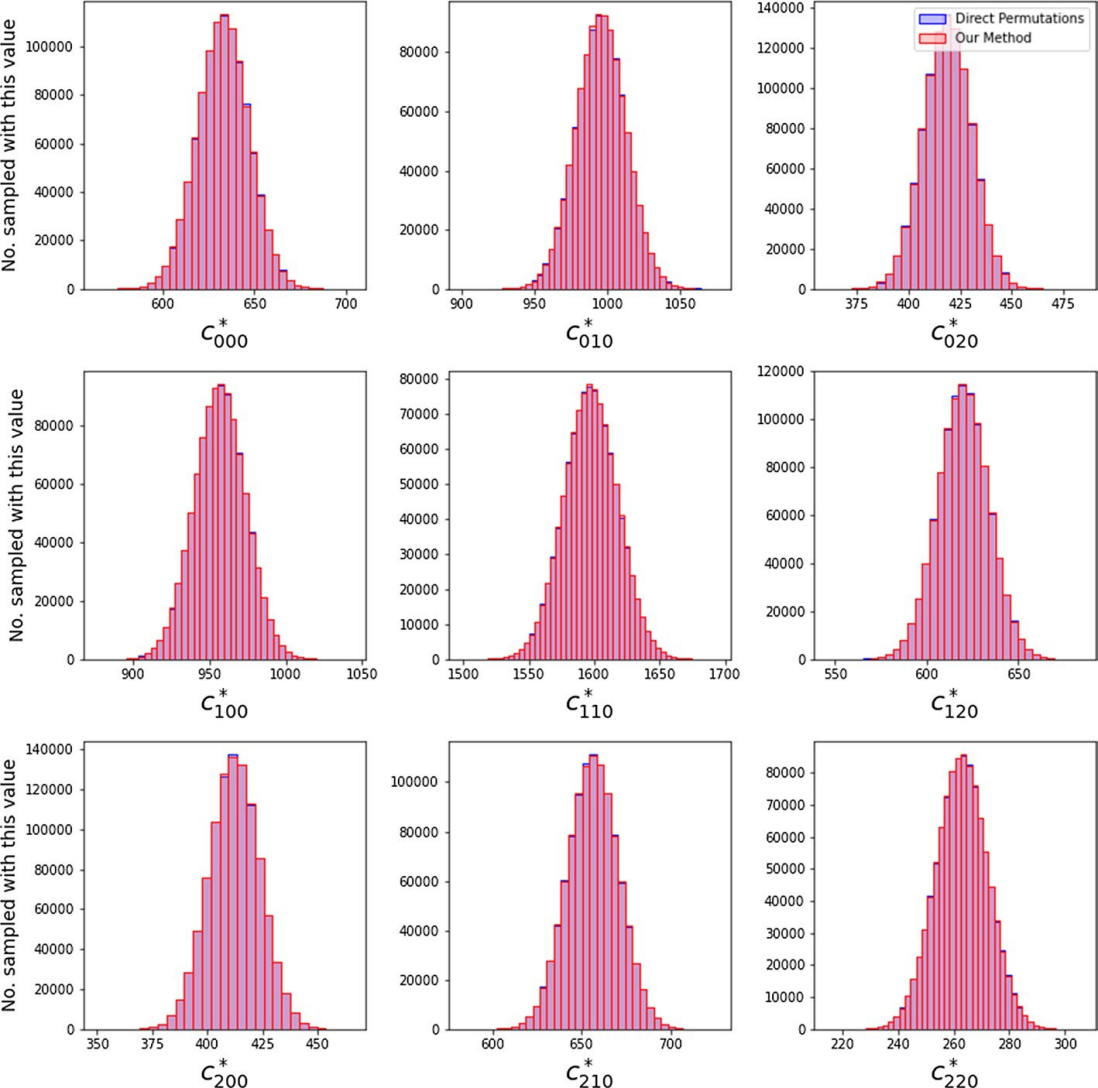
Using the simulated data described in Sect. , we generated 1,000,000 permuted count tables using both the naive method of directly permuting the phenotype labels and using our approach. The distributions of the count table elements $c_{ij0}^*$ are plotted here, with the direct permutation result shown in blue and our method shown in red. The plot consists almost entirely of the purple overlapping region, as there is almost no visible difference between the distributions

We verify that our method is working as desired by permuting the above count table $N_P=1{,}000{,}000$ times using two different approaches: (1) the naive permutation testing approach, in which we randomly shuffle the phenotype vector and re-compute the count table; (2) our method as outlined in Sect.  of the main text. The distributions of the elements $c_{ij0}^*$ are shown in Fig. 2. As shown in the figure, the resulting distributions are nearly identical, and the distributions generated from the two approaches overlap almost perfectly. The computational cost savings of this approach are considerable. On our machine, generating $N_P=1{,}000{,}000$ permuted count tables took a total of 761.8 seconds using the naive method and only 5.7 seconds using our method.

It is immediately obvious from Fig. 2 that the distributions are very close to normal distributions, which is not surprising given our relatively large choice of $N_P$. One may be tempted to use this fact to formulate a simpler approach to generating random count tables: could we simply estimate the normal distributions for each $c_{ij0}^*$ and sample those directly? This approach will not work because the elements are not independent from each other, meaning that an iterative procedure such as ours is required.

### Distributions of information measures

Having generated an ensemble of 1,000,000 count tables using each method, we can compute the joint entropies of our variables as well as any information theoretic measure which is a function of the entropies. For example, we can compute the multi-information:

9 $$\begin{aligned} \Omega = -H_{123} + \sum _i H_i \end{aligned}$$

where $H_i$ are the entropies of each individual variable, and $H_{123}$ is the joint entropy of all three variables (i.e. our two SNPs and the phenotype).

**Fig. 3:**
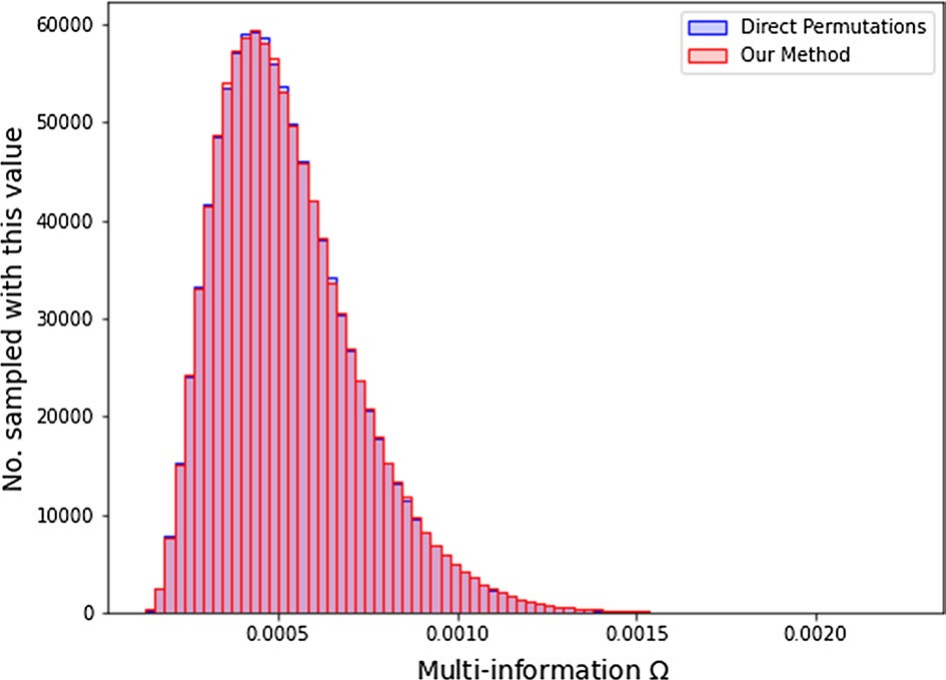
The permuted count tables from Sect.  can be used to calculate the joint entropies, from which we can calculate any information theoretic measure which is a function of the entropies. Here we calculate the multi-information $\Omega$ using both the count tables generated by direct permutations and by our method, with the resulting distributions being nearly identical

The subsequent computation of information measures is considerably less expensive than the construction of the count tables. For instance, computing the distributions of $\Omega$ values using either set of the 1,000,000 count tables generated in the previous section took 1.6 s. Figure 3 shows the distributions of $\Omega$ values based on the count tables generated by two different permutation methods in the previous section. Once again, we see that our method yields a nearly identical distribution to the naive method of direct permutation. In the case of real data analysis, these permuted distributions would serve as null distributions in our significance analysis. This result verifies that our method produces a null distribution equally sufficient for significance analysis as the naive permutation method, but at considerably less computational expense.

### Statistical testing of distribution equivalence

The distributions in Figs. 2 and 3 appear to be nearly identical, but we wish to test (1) whether or not they may be distinguished via statistical testing, and (2) whether or not this result is sensitive to the choice of parameters *p* and *q*. We therefore ran 1000 trials of the following:

1. Independently choose parameter values *p*, *q* from a uniform random distribution on (0.01, 0.99), and use this to generate a count table with $n=10{,}000$ samples.
2. Generate $N_P=1000$ permuted count tables using both direct permutation of phenotype labels and our method.
3. For each $c_{ij0}^*$, perform a two-sample Epps–Singleton test comparing the two methods.

**Fig. 4:**
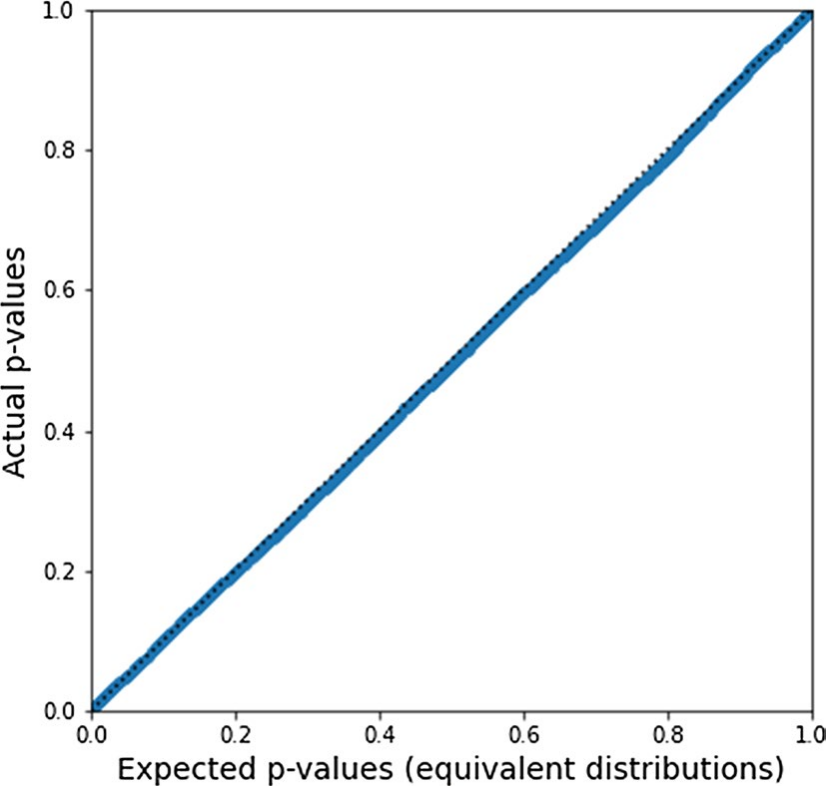
Quantile–quantile plot of *p* values from Epps–Singleton tests comparing the two distributions. The null hypothesis is that the two distributions are identical. Under the null hypothesis, the *p* value is uniformly distributed and we would expect the Q–Q plot to be linear along the diagonal, which is what we observe. The count table distributions generated from each method are indistinguishable via this test

This will yield 9000 *p* values generated under a broad range of different parameter values. The Epps–Singleton test [39] has the null hypothesis that both samples are drawn from the same distribution (and is used here since it allows for discrete distributions). By definition, the *p* values should be uniformly distributed under the null hypothesis. In Fig. 4, we show that our *p* values are fully compatible with a uniform distribution, such that the count tables generated by naive permutation and those generated by our method are not statistically distinguishable.

## Data Availability

The datasets generated and/or analysed during the current study are available in the Zenodo repository, http://doi.org/10.5281/zenodo.4068765

## Abbreviations

GWAS: Genome-wide association study
SNP: Single nucleotide polymorphism
